# Immune Cell-Derived Extracellular Vesicles in the Face of Pathogenic Infections

**DOI:** 10.3389/fimmu.2022.906078

**Published:** 2022-06-30

**Authors:** Somayeh Keshtkar, Saeede Soleimanian, Maryam Kaviani, Fatemeh Sabet Sarvestani, Negar Azarpira, Zahra Asvar, Sara Pakbaz

**Affiliations:** ^1^Autophagy Research Center, Shiraz University of Medical Sciences, Shiraz, Iran; ^2^Molecular Dermatology Research Center, Shiraz University of Medical Sciences, Shiraz, Iran; ^3^Transplant Research Center, Shiraz University of Medical Sciences, Shiraz, Iran; ^4^Allergy Research Center, Shiraz University of Medical Sciences, Shiraz, Iran; ^5^Nanotechnology School of Advanced Medical Sciences and Technologies, Shiraz University of Medical Sciences, Shiraz, Iran; ^6^Department of Laboratory Medicine and Pathobiology, Faculty of Medicine, University of Toronto, Toronto, ON, Canada; ^7^Department of Laboratory Medicine & Pathobiology, Mount Sinai Hospital, Toronto, ON, Canada

**Keywords:** extracellular vesicles, immune cell, microbial infection, anti-microbial, pathogens

## Abstract

Extracellular Vesicles (EVs) are a collection of vesicles released from cells that play an important role in intercellular communication. Microbial infections are known as one of the major problems in the medical field. Considering the increasing resistance of strains to routine drug treatments, the need for new therapies seems to be more than ever. Recent studies have shown that the EVs released from immune cells during microbial infections had anti-microbial effects or were able to induce neighbouring cells to display anti-microbial effects. This mini-review aimed to explore the latest studies on immune cell-derived EVs in viral, bacterial, fungal, and parasitic infections. Review of the literature demonstrated that specific cargos in EVs were involved in the fight against pathogenic infections. Additionally, the transport of appropriate bioactive molecules including miRNAs, mRNAs, and proteins *via* EVs could mediate the anti-microbial process. Thus, it could be a proof-of-principle that therapeutic approaches based on EVs derived from immune cells could offer a promising path forward, which is still in early stages and needs further assessments.

**Graphical Abstract:**
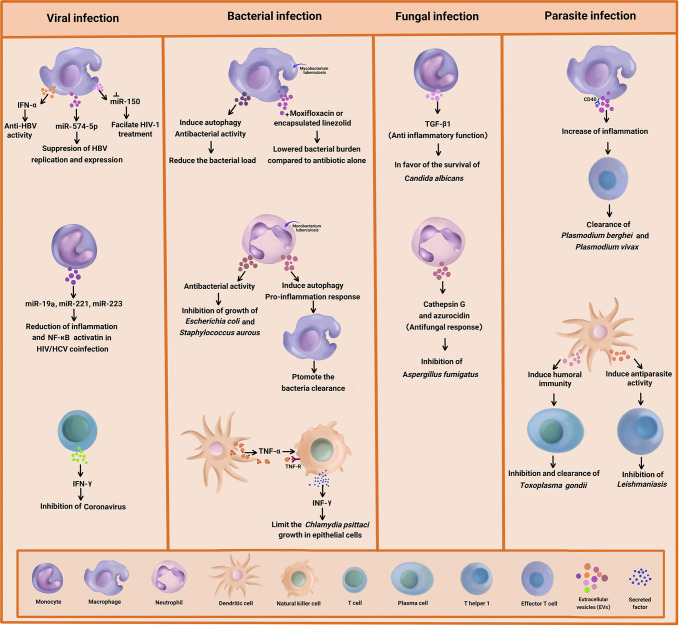
Summary of the antimicrobial effect of immune cell-derived extracellular vesicles (EVs) on bacteria, viral, fungal, and parasitic diseases.

## Introduction

The immune system is a complex system consisting of different cell types that reside in multiple organs throughout the body. The connection pathways in the immune system develop by direct contacts between these cells and the release of soluble factors to maintain cellular homeostasis and host defense (1). Raposo et al. introduced a new means of communication based on the release of substances into the extracellular space called Extracellular Vesicles (EVs) (2). More than 50 years ago, Wolf et al. discovered EVs in plasma as “platelet dust” (3). EVs include a heterogeneous group of membrane-bound particles present in all biological fluids. Based on their mechanism of release and size, EVs have been categorized as a) exosomes (diameter < 150 nm), b) microvesicles/shedding particles (150-1000 nm), and c) apoptotic bodies (> 150 nm) (2, 4, 5). In contrast to microvesicles that are secreted by budding from the cell membrane, exosomes originate as intraluminal vesicles in multivesicular endosomes and fuse with the plasma membrane to release into the extracellular space. Exosome biogenesis depends on various critical factors including the site of biogenesis, protein sorting, physicochemical aspects, and transacting mediators. Endosomal Sorting Complex Required for Transport (ESCRT), exosome-bound proteins, annexins, and Rab protein govern membrane transport and fusion, whereas Alix, flotillin, and TSG101 are involved in exosome biogenesis (6–10).

Heterogeneity in size, content and source of EVs can affect on recipient cells responses such as cell survival, death, inflammation and immune response, or assist pathogens to enter and stay in the recipient cell (11). Also, EVs are isolated based on size by gradient ultracentrifugation, coprecipitation, and immunoaffinity enrichment method (12). Moreover, their characterization methods include mass spectrometry, western blot, immunoelectron microscopy image, fluorescence nanoparticle tracking analysis, and polychromatic flow cytometry based on the molecules expressed on their surface (13).

Immune cell-derived EVs are enriched in proteins, especially tetraspanins. Tetraspanins refer to a family of ubiquitous proteins that include CD9, CD63, CD81, and CD82. These proteins on EVs interact with other proteins such as Major Histocompatibility Complex (MHC) molecules and integrins expressed on target cells, eventually leading to prime-specific immune responses (14, 15). They also exhibit special lipid compositions in significant quantities, including sphingomyelin, phosphatidylcholine, and phosphatidylethanolamine (16–19). Immune cell-derived EVs also contain a unique set of enzymes that are involved in lipid metabolism (phospholipase A2, C, and D) (16, 20). Immune cell-derived EVs alter many physiological and pathological processes. In addition, they play important roles in innate and acquired immune responses including antigen presentation, immune cells maturation, differentiation, activation, and suppression, and anti-inflammatory and anti-microbial effects (21–23). Moreover, EVs from host cells can participate in anti-microbial immunity *via* the production and release of Interferon (IFN), IL-12, granzyme B, perforin, non-coding RNA, etc. (24–26).

The first reports pertaining to functionally active immune cell-derived EVs were provided by Raposo et al. (27). They showed that B cell-derived EVs could stimulate antigen-specific T cell responses *via* functional peptide–MHC II complexes. Antigen presentation usually needs Antigen Presenting Cells (APCs), which process antigenic peptides and form MHC-peptide complexes. After that, this complex binds to T cell receptors with the synergism of co-stimulatory molecules to activate and proliferate T cells. However, EVs, especially exosomes, make the host’s immune system stronger against invading pathogens without the interaction between APCs and T cells and even without the reprocessing of MHC-peptide complex by the recipient APCs. This results from the fact that they are enriched in efficient carriers of MHC II-peptide complexes and other molecules, allowing robust T cell activation (22, 28–33). What follows includes the investigation of the role of immune cell-derived EVs in combating pathogens.

## Immune Cell-Derived Extracellular Vesicles in Viral Infection Therapy

Generally, a variety of virus-infected cells release EVs that are related to the pathogenesis, diagnostics, and therapeutics of viral diseases. It is noteworthy that immune cell-derived EVs play an important role in the treatment of viral infections. The review of the literature revealed evidence for the EVs derived from monocytes, macrophages, and T cells in the treatment of viral diseases.

Different types of Micro RNA (miRNA) are present in EVs, which are involved in the regulation of cell to cell communication. The cytosol expression of miR-29a and miR-150 was previously reported (1). Further studies indicated strongly upregulated miR-29a and miR-150 in the HIV-Infected macrophages (2). The potential of miR-150 involvement in the HIV/AIDS disease progression and therapy has been elucidated, as well. Accordingly, miR-150 suppression might facilitate HIV-1 treatment (34).

Researchers investigated the role of macrophage-derivedexosomes in the inhibition of DNA replication in Hepatitis BVirus (HBV). They analyzed the expression of different exosomal miRNA and suggested that they were closely related to liver inflammation injury and viral replication (35). In another research, miR-638 was found to target the regulation of a variety of cellular processes such as proliferation, apoptosis, and inflammation (36).

The induction of the macrophages using INF can result in the production of exosomes mediating antiviral activity against HBV. This phenomenon might be associated with the transfer of miR-574-5p from macrophages to HBV-infected hepatocytes, leading to the suppression of HBV replication and expression (37). On the other hand, macrophage-derived exosomes can exert their antiviral properties by transferring IFN-α to infected cells. In this regard, Yao et al. reported that the exosomes derived from macrophages induced anti-HBV activity through the delivery of IFN-α to hepatocytes. The exosomes used T cell immunoglobulin and mucin receptor 1 to enter the hepatocytes (38).

A recent study investigated the reduction of inflammation in Hepatitis C Virus (HCV) treatment amongst HIV/HCV-coinfected individuals. The results indicated that in the coinfected people, miR-19a, miR-221, and miR-223 were upregulated in the monocyte-derived exosomes, which were related to the reduction of plasma inflammation and activation of Nuclear Factor-κB (NF-κB) in the liver (6). Another research showed that miR-221 triggered the anti-inflammatory cascade in monocytes (39) and hindered HIV-1 entry into macrophages by targeting the CD4 viral receptor (40).

It has been reported that miR-19a is involved in the restoration of monocyte immune functions in the coinfection of HIV/HCV (41). miR-223 has been identified as an anti-inflammatory microRNA which acts through the NF-κB pathway (42). The NF-κB pathway has long been regarded as a prototypic pro-inflammatory signalling pathway. Pro- and anti-inflammatory roles of this pathway have been underlined in recent studies. Accordingly, NF-kB exerted anti-inflammatory effects through the direct inhibition of pro-inflammatory genes as well as impacts on the expression or activity of anti-inflammatory cytokines (43).

Up to now, limited studies have been conducted on anti-viral responses through T cell-derived EVs. T cell-derived exosomes could prime Dendritic Cells (DCs) through their cargos, which are genomic and mitochondrial DNA. These factors triggered antiviral responses *via* the cGAS/STING cytosolic DNA-sensing pathway as well as the expression of Interferon Regulatory Factor 3 (IRF3)-dependent genes (44). T cell-derived exosomes have also been proposed for the treatment of COVID-19 infection. In a single-arm, open-labeled, combined interventional (phase I/II trials) clinical trial (NCT04389385), the safety and efficacy of T cell-derived exosomes in the treatment of COVID-19 infection was explored. In that project, specific T cells from COVID-19 were activated and expanded through exposure with viral peptide fragments and cytokines. The results suggested that mediators like IFN-gamma in the secreted exosomes from T cells might inhibit the coronavirus (45).

Overall, the literature review provided proof-of-principle for the application of immune cell-derived EVs in anti-viral strategies. Accordingly, specific cargos in exosomes were involved in the fight against viral infections, and the transport of appropriate bioactive molecules including miRNAs *via* exosomes could mediate the antiviral process.

## Immune Cell-Derived Extracellular Vesicles in Bacterial Infection Therapy

One of the new approaches in the diagnosis, pathogenesis, and treatment of bacterial infections is the use of EVs separated from infected immune cells. EVs released from infected immune cells have shown anti-bacterial effects on pathogenic bacteria. Neutrophil-derived EVs, in particular, are one of the most important innate immune cells for controlling bacterial infections (46). Timar et al. investigated the effects of EVs derived from human Neutrophilic Granulocytes (PMNs) in response to *Staphylococcus aurous* infection (47). They came to the conclusion that the pre-stimulation of PMNs by various agents induced the release of EVs with different biological properties. Accordingly, only the PMNs stimulated with opsonized particles produced EVs that had the ability to impair bacterial growth. Moreover, the anti-bacterial effect of PMN-derived EVs was correlated to the aggregation of bacteria on their surface, which depended on intact cytoskeleton and metabolic activity within the vesicles. Nonetheless, the anti-bacterial activity of EVs in response to *Staphylococcus aurous* infection was not limited to these bacteria, as the growth of *Escherichia coli* was inhibited by neutrophil-derived EVs, as well. However, this anti-microbial activity had no effects on *Proteus mirabilis* infections, suggesting some levels of specificity on different types of bacteria (32, 47). Interestingly, the anti-bacterial activity of neutrophil-derived EVs depended on particles/bacterial opsonisation through the activation of PLCϒ2 and opsonin receptors as well as the presence of extracellular calcium and Mac-1 integrin complex, independent of the phagocytic process (48, 49).

In another study, EVs derived from neutrophils infected with *Mycobacterium tuberculosis* (Mtb) activated macrophages and promoted the clearance of intracellular Mtb *via* enhancing early superoxide anion production and autophagy induction (50). This suggested that EVs acted indirectly by promoting the immune response in neighbouring cells. In an interesting study by Garcia-Martinez et al., EVs released from J774A.1 mice’s macrophage cell line mitigated the bacterial load and production of MCP-1 and TNF-α cytokines in a phosphatidylserine-dependent manner in Mtb-infected macrophages, which suggested the anti-bacterial activity of EVs including exosomes. Furthermore, *in vivo* results indicated the reduction of the lung bacterial load by macrophage-derived EVs (51).

The mechanism of the anti-bacterial action of EVs is not entirely clear. EVs may be able to capture pathogen derived nucleic acid and protein and transport them to host cell and trigger anti-bacterial immune activity. The results of the research carried out by Cheng and Schorey demonstrated that the transport of *Mycobacterium* RNA to EVs released from infected macrophage led to the activation of the host RIG-I/MAVS/TBK1/IRF3 RNA sensing signaling and the production of type I INF in recipient cells (52). They showed that the present of pathogen RNA in EVs is dependent on the bacteria’s SecA2 secretion system, suggesting the intercellular transfer of bacterial RNA through host cell-derived EVs may also be perceived for other pathogens that express a SecA2 secretion system such as *Staphylococcus, Listeria, and Streptococcus* species (53). Moreover, EVs released from Mtb-infected macrophages induced autophagy *via* LC3-associated pathway and enhanced bacterial killing. Indeed, LC3-associated pathway illustrates an autophagy-dependent antimicrobial pathway in host cells that led to increasing microbial degradation (54). Furthermore, the combination of moxifloxacin and EVs isolated from Mtb-infected macrophages notably lowered the bacterial burden compared to drug treatment alone (52), suggesting a new immunotherapeutic approach to treat drug-resistant Mtb.

Today, drug delivery systems at the nanoscale take up considerable space. Various formulations of nanomedicines have been used to enhance the therapeutic efficacy of chemical and biomolecular medicines. EVs have appeared to be biocompatible vehicles for the delivery of various drugs including antibiotics. Yang et al. disclosed that macrophage-derived EVs with encapsulated linezolid antibiotics were more effective against Methicillin-Resistant *Staphylococcus aureus* (MRSA) infections in comparison to free linezolid antibiotics (55). Thus, EVs derived from immune cells were found to be biocompatible vehicles for the delivery of various drugs including anti-microbial agents.

More recently, it was demonstrated that EVs released from *Chlamydia psittaci*-infected DCs were strongly able to induce INF-γ production and secretion in natural killer cells through a TNF-α/TNF receptor interaction (56). The combination of EVs with INF-γ and TNF-α released from infected DCs and neighbouring NK cells limited the *C. psittaci* growth in infected epithelial cells and attenuated the subversion bacterial resistance to apoptosis. Overall, that study emphasized that the induction of pro-inflammatory cytokines by EVs derived from host immune cells could further activate the anti-bacterial defense (56). A similar mechanism was also applied by the host to deliver cytokines for the activation of immune response in combat with infections. Accordingly, DC-derived EVs transferred the pro-inflammatory cytokine TNF-α and activated the neighbouring epithelial cells, leading to the release of additional inflammatory cytokines and chemokines as well as the promotion of the innate immune response. All in all, these limited studies highlighted the role of immune cell-derived EVs in response to bacterial infections. Yet, more detailed studies in this area are warranted.

## Immune Cell-Derived Extracellular Vesicles in Fungal and Parasitic Infection Therapy

The immune system is concomitantly involved with infectious agents such as fungi and parasites to generate exosomes that are applicable in defense against these pathogens. Hence, immune cell-derived EVs could be considered a valuable treatment target against these infectious agents. In this regard, investigations were done on *Aspergillus fumigatus* and *Candida albicans*, as two clinically relevant’ human fungal pathogens, to explore the host’s immune response (57). Based on the findings, anti-fungal responses from neutrophil-produced EVs were shown in response to *A. fumigatus* infection. Indeed, *A. fumigatus* stimulates EV produced by human neutrophils and utilizing these EVs *in vitro* led to the elimination of fungal hyphae (58, 59). As such, during infection with opsonized A.fumigatus, fresh human polymorph nuclear granulocytes(PMNs) were extracted, and then EV release by human neutrophils were collected and co-incubated with fungi for 4 to 6 hour at a different multiplicity of infection (MOI) to one PMNs to optimize minimal cell death in the PMN population (60). Accordingly, the EVs could attach to and enter fungal conidia and inhibit their growth (57). Surprisingly, anti-fungal cargo proteins including cathepsin G and azurocidin were identified in these anti-fungal EVs through mass spectrometry-based proteomic analysis. Notably, these productions from the host were specifically affirmed against a mutant strain of A. fumigatus (61). Conversely, a recent study found that monocyte-derived EVs presented anti-inflammatory functions in the context of *C. albicans* infection. TGF-β1-transporting EVs, as anti-inflammatory vesicles, were released *via* interaction between Complement Receptor 3 (CR3, also known as CD11b/CD18) on monocytes and soluble β-glucan produced from *C. albicans* (62). In addition, TGF-β1 led to the development of immune modulation, favoring the survival of C. *albicans* commensalism (63). Inconsistent with the anti-fungal role of EVs in *A. fumigatus* infection, these EVs appeared to provide an opportunity for *C. albicans* to be a human commensal. It is noteworthy that the lipid-enclosed Amphotericin B (AmBisome) was utilized instead of amphotericin B, which enhanced the uptake and decreased the off-target effects (64). This technique is classically similar to transfer *via* EVs. Overall, these findings proved a promising path forward to target exosomes for fungal pathogens of interest (65, 66).

Plasmodium berghei, as a rodent malaria parasite, leads to the secretion of plasma cell-derived EVs and stimulates antigen-presenting cells through CD40, which generates an inflammatory response that causes the initiation of effector T cells activity (67). Therefore, macrophage induction triggers the parasite clearance. Evidence has indicated that the distinct properties of EVs make them of interest for new drugs and treatment prospects in parasitic infections. Hence, attention has to be paid to the potential of exosomes as a therapeutic agent and vaccine target in parasitic diseases.

Conversely, during acute Plasmodium vivax infection in humans, the presence of increased circulating immune cell-derived EVs might be important on the acute inflammatory signs of malaria vivax (68, 69). Besides, EVs derived from Toxoplasma gondii antigen-pulsed DCs could be candidate as an effective vaccine against toxoplasmosis, because these pulsed DCs were able to induce humoral immunity against the parasite (70). This was further supported by a study performed on cutaneous leishmaniasis, which indicated that protective Th1 responses were mediated by DC-derived EVs (71). Similar results were also obtained regarding common livestock parasites, which showed that the EVs derived from parasite antigen-loaded DCs played an important role in protection against the infection (72). This could provide a proof-of-principle that therapeutic approaches based on EVs derived from immune cells offer a promising path forward.

Considering the results of earlier studies pertaining to the release of EVs from parasites or parasitized cells, utilizing suitable strategies against the function of these EVs can be a therapeutic approach. Accordingly, there are particular interactions between parasite-derived EVs and the immune system (73). Another support for the therapeutic application of exosomes in parasitic diseases comes from a study by Chappuis et al. (74), which described challenges in the treatment of visceral leishmaniosis including drug resistance and variable responses to treatment regimens, leading to a long sought treatment. Nonetheless, leishmania exosomes were found to affect the innate and adaptive immune responses (75, 76). In this regard, designing immune cell-derived EVs containing the related drugs or specific cargoes may lead to the activation and induction of immune responses, inducing protection against parasitic diseases. This technique can be used to treat such infections, and such an insight will be highly valuable.

## Conclusion

The studies reported in this mini-review demonstrated that the EVs released by immune cells were able to invade various pathogens including bacterial, viral, and fungal infections. However, unanswered questions have remained about the antimicrobial effects of EVs including their exact mechanism of action. On the other hand, the exact cargos of EVs produced by each immune cell are changeable depending on the pathophysiology of the microenvironment. Thus, valuable information can be gained through a close evaluation of the role of EVs during infections. Hopefully, the years to come will witness a change in the use of EVs as both diagnostic and therapeutic agents for the treatment of infectious diseases.

## Author Contributions

All authors listed have made a substantial, direct, and intellectual contribution to the work, and approved it for publication.

## Conflict of Interest

The authors declare that the research was conducted in the absence of any commercial or financial relationships that could be construed as a potential conflict of interest.

## Publisher’s Note

All claims expressed in this article are solely those of the authors and do not necessarily represent those of their affiliated organizations, or those of the publisher, the editors and the reviewers. Any product that may be evaluated in this article, or claim that may be made by its manufacturer, is not guaranteed or endorsed by the publisher.
